# Progressive demyelinating polyneuropathy after hematopoietic cell transplantation in metachromatic leukodystrophy: a case series

**DOI:** 10.1007/s00415-024-12322-3

**Published:** 2024-04-02

**Authors:** Shanice Beerepoot, Jaap Jan Boelens, Caroline Lindemans, Moniek A. de Witte, Stefan Nierkens, Alexander F. J. E. Vrancken, Marjo S. van der Knaap, Marianna Bugiani, Nicole I. Wolf

**Affiliations:** 1 Amsterdam UMC, Department of Child Neurology, Amsterdam Leukodystrophy Center, Emma’s Children’s Hospital, VU University, Amsterdam, The Netherlands; 2 Neuroscience, Cellular & Molecular Mechanisms, VU University, Amsterdam, The Netherlands; 3 Center for Translational Immunology, University Medical Center Utrecht, Utrecht, The Netherlands; 4 Princess Máxima Center for Pediatric Oncology, Utrecht, The Netherlands; 5 Department of Pediatrics, Stem Cell Transplant and Cellular Therapies, Memorial Sloan Kettering Cancer Center, New York, NY USA; 6 Regenerative Medicine Institute, University Medical Center Utrecht, Utrecht, The Netherlands; 7 Department of Hematology, University Medical Center, Utrecht, The Netherlands; 8 Department of Neurology, Brain Centre University Medical Center Utrecht, Utrecht University, Utrecht, The Netherlands; 9 Department of Functional Genomics, Center for Neurogenomics and Cognitive Research, VU University, Amsterdam, The Netherlands; 10 Amsterdam UMC, Department of Pathology, VU University Amsterdam, Amsterdam, The Netherlands

**Keywords:** Metachromatic leukodystrophy, *ARSA* gene mutation, Lysosomal storage disorder, Demyelinating polyneuropathy, Hematopoietic cell transplantation, Immune-mediated demyelinating disease

## Abstract

Metachromatic leukodystrophy (MLD) is a neuro-metabolic disorder due to arylsulfatase A deficiency, causing demyelination of the central and peripheral nervous system. Hematopoietic cell transplantation (HCT) can provide a symptomatic and survival benefit for pre-symptomatic and early symptomatic patients by stabilizing CNS disease. This case series, however, illustrates the occurrence of severely progressive polyneuropathy shortly after HCT in two patients with late-infantile, one with late-juvenile, and one with adult MLD, leading to the inability to walk or sit without support. The patients had demyelinating polyneuropathy before HCT, performed at the ages of 2 years in the first two patients and at 14 and 23 years in the other two patients. The myeloablative conditioning regimen consisted of busulfan, fludarabine and, in one case, rituximab, with anti-thymocyte globulin, cyclosporine, steroids, and/or mycophenolate mofetil for GvHD prophylaxis. Polyneuropathy after HCT progressed parallel with tapering immunosuppression and paralleled bouts of infection and graft-versus-host disease (GvHD). Differential diagnoses included MLD progression, neurological GvHD or another (auto)inflammatory cause. Laboratory, electroneurography and pathology investigations were inconclusive. In two patients, treatment with immunomodulatory drugs led to temporary improvement, but not sustained stabilization of polyneuropathy. One patient showed recovery to pre-HCT functioning, except for a Holmes-like tremor, for which a peripheral origin cannot be excluded. One patient showed marginal response to immunosuppressive treatment and died ten months after HCT due to respiratory failure. The extensive diagnostic and therapeutic attempts highlight the challenge of characterizing and treating progressive polyneuropathy in patients with MLD shortly after HCT. We advise to consider repeat electro-neurography and possibly peripheral nerve biopsy in such patients. Nerve conduction blocks, evidence of the presence of T lymphocytes and macrophages in the neuronal and surrounding nerve tissue, and beneficial effects of immunomodulatory drugs may indicate a partially (auto)immune-mediated pathology. Polyneuropathy may cause major residual disease burden after HCT. MLD patients with progressive polyneuropathy could potentially benefit from a more intensified immunomodulatory drug regime following HCT, especially at times of immune activation.

**Supplementary Information:**

The online version contains supplementary material available at 10.1007/s00415-024-12322-3.

## Introduction

Metachromatic leukodystrophy (MLD, OMIM #250,100) is an inherited lethal neurometabolic disorder caused by deficiency of the lysosomal enzyme arylsulfatase A (ASA) [1]. ASA catalyzes desulfation of 3-O-sulfogalactosyl residues (sulfatides) in glycosphingolipids, and its deficiency results in intralysosomal sulfatide accumulation [2]. Myelin sheaths of the central and peripheral nervous system are predominantly affected, leading to progressive demyelination and, to a lesser extent, axonal loss [3, 4]. The most prominent clinical features are motor and cognitive regression, ataxia, pyramidal signs, and eventually loss of all motor function and speech [5, 6]. Based on the age of disease onset, four clinical types of MLD can be distinguished, including late-infantile (< 2.5 years), early-juvenile (2.5–6 years), late-juvenile (6–16 years), and adult (> 16 years). Generally, the younger the age of onset, the faster the disease progression [7–9].

Allogeneic hematopoietic cell transplantation (HCT) can provide a symptomatic and survival benefit for presymptomatic and early symptomatic patients with MLD [10, 11]. However, progressive polyneuropathy may cause major disease burden, despite otherwise successful HCT [12]. Our systematic review indicates that approximately 75% of the HCT-treated patients show a decline in nerve conduction velocity (NCV) or deterioration of clinical symptoms [12], but information about detailed clinical course of peripheral polyneuropathy progression after HCT is scarce, and its cause and pathology remain unclear. We wondered whether progressive polyneuropathy after HCT should only be attributed to ongoing sulfatide accumulation, especially in case of rapid deterioration shortly after treatment. Alternative causes include neurological toxicity of HCT drugs, graft-versus-host disease (GvHD) or another (auto)immune-mediated cause [13–16]. This case series illustrates progressive polyneuropathy in two patients with late-infantile (MLD-45 and MLD-50), one with late-juvenile (MLD-87), and one with adult MLD (MLD-62), exemplifying the diagnostic and therapeutic challenges in these patients.

## Methods

For this patient record review study, 4 subjects with a confirmed diagnosis of MLD were included in the Amsterdam Leukodystrophy Center, a Dutch nationwide expertise center [17]. The patients were selected because their polyneuropathy progressed significantly within 1 year post-HCT despite the fact that HCT was performed early in the disease course, and are derived from a cohort of 18 patients, the majority diagnosed with demyelinating polyneuropathy, who have received HCT since 2004 in the same center. Significant progression of polyneuropathy was defined as an increase in muscle weakness, whether accompanied by new signs or symptoms indicating impaired sensory function and the absence of deep tendon reflexes, leading to considerable disability when compared to the pre-HCT status. HCT outcomes other than polyneuropathy from part of this cohort, including patients MLD-45 and MLD-50, were described previously [10, 18]. The local Institutional Review Board approved the study, and appropriate written consent was obtained according to the Declaration of Helsinki.

## Results

The four patients experiencing severe and rapidly progressing polyneuropathy shortly after HCT, which significantly contributed to their inability to walk or sit without support, are described in detail in the supplementary material. In summary, all had signs of a severe demyelinating sensorimotor polyneuropathy on neurological examination and nerve conduction studies before HCT performed at the ages of two years in patients MLD-45 and MLD-50, 14 years in MLD-87, and 23 years in MLD-62. Cognitive function was normal for age in MLD-45, MLD-50, and MLD-62 and below average but in line with school level and stable in MLD-87. An overview of patient characteristics, test results, and treatment details before and after HCT is presented in Table 1. All patients achieved full-donor chimerism in blood at 1 month post-HCT. Post-HCT course was complicated by multiple viral infections, acute-GvHD varying from grade 1 (MLD-45 and MLD-50) to grade 3 (MLD-62), diabetes dysregulation (only in MLD-62), and slow restitution of T lymphocytes (only in MLD-45).

**Table 1:** Patient characteristics, HCT details, and follow-up data

	MLD-45	MLD-50	MLD-62	MLD-87
Sex	Female	Male	Male	Female
Relevant medical history	Premature birth, never achieved independent walking	Never achieved independent walking	Poorly controlled type 1 diabetes	General clumsiness, slightly reduced fine motor skills, MPNST
MLD type	Late-infantile	Late-infantile	Adult	Late-juvenile
Age at				
Presentation	17 months	23 months	22 years	12 years
Diagnosis	23 months	25 months	23 years	14 years
*ARSA* mutations				
Allele 1	c.465 + 1G > A (r.0)	c.251C > T (p.Pro84Leu)	c.1283C > T (p.Pro428Leu)	c.1150G > A (p.Glu384Lys)
Allele 2	c.830_831delTCinAA (p.Ile277Lys)	c.1174C > T (p.Arg392Trp)	c.1283C > T (p.Pro428Leu)	c.692A > C (p.His231Pro)
Residual ASA activity at diagnosis	4 nmol/17 h/mg protein	Undetectable	6 nmol/17 h/mg protein	23 nmol/17 h/mg protein
Urinary sulfatide excretion	N.A.	N.A.	301 nmol/mmol creatinine	360 nmol/mmol creatinine
Symptoms before HCT	Delayed gross motor milestones, strabismus, ptosis	Progressive walking difficulties with foot drop and hypotonia	Sensory abnormalities and mild foot drop	Coordination and balance difficulties, mild foot drop, incontinence, bilateral tremor and pyramidal signs
Cognitive functioning before HCT (test)	MDI = 101 (BSID-II-NL)	MDI = 111 (BSID-II-NL)	IQ = 99 (WAIS-III-NL)	IQ = 72 (WISC-V-NL)
Brain MRI before HCT	Mildly delayed myelination and subtle nonspecific white matter abnormalities, MLD-LOES score: 3	Mildly delayed myelination, MLD-LOES score: 2	Extensive white matter abnormalities and mild global atrophy, MLD-LOES score: 12	White matter abnormalities and mild global atrophy, MLD-LOES score: 11
Nerve conduction velocity at diagnosis				
Median nerve (sensory)	No response	No response	N.A.	61 m/s
Ulnar nerve (sensory)	No response	N.A.	No response	23 m/s
Sural nerve (sensory)	N.A.	N.A.	No response	N.A.
Median nerve (motor)	No response	15 m/s	N.A.	19 m/s
Ulnar nerve (motor)	No response	N.A.	23 m/s	22 m/s
Peroneal nerve (motor)	6 m/s	N.A.	12 m/s	15 m/s
Tibial nerve (motor)	16 m/s	N.A.	15 m/s	23 m/s
Distal motor latency at diagnosis				
Median nerve (motor)	10 ms	9 ms	N.A.	6 ms
Ulnar nerve (motor)	9 ms	N.A.	5 ms	6 ms
Peroneal nerve (motor)	7 ms	N.A.	16 ms	8 ms
Tibial nerve (motor)	9 ms	N.A.	19 ms	N.A.
Age at HCT	25 months	27 months	23 years	14 years
HCT conditioning				
Fludarabine	160 mg/m^2^	160 mg/m^2^	160 mg/m^2^	160 mg/m^2^
Busulfan (targeted)	90 mg*h/L	90 mg*h/L	90 mg*h/L	90 mg*h/L
Rituximab				375 mg/m2
GvHD prophylaxis in starting dose				
ATG	10 mg/kg	10 mg/kg	7.5 mg/kg	12 mg/kg
Prednisone	1 mg/kg/day	1 mg/kg/day	N.A.	1 mg/kg/day
Cyclosporine (targeted)	200–250 mg/L/day	200–250 mg/L/day	200–250 mg/L/day	200–250 mg/L/day
Mycophenolate mofetil	50 mg/kg/day (after cyclosporine treatment)	50 mg/kg/day (initiated 9 months post-HCT)	50 mg/kg/day (concurrent with cyclosporine treatment)	
Donor details				
Source	Cord blood (unrelated)	Cord blood (unrelated)	Bone marrow (unrelated)	Cord blood (unrelated)
HLA matching	6 out of 6	6 out of 6	10 out of 10	6 out of 6
Engraftment (absolute neutrophil count > 500)	17 days post-HCT	14 days post-HCT	40 days post-HCT	14 days post-HCT
Donor chimerism after HCT				
One month	97%	97%	99%	98%
Latest FU (FU in years)	100% (3)	N.A.	100% (1)	100% (2)
ASA activity after HCT				
Two months	128 nmol/17 h/mg protein	87 nmol/17 h/mg protein	N.A.	N.A.
Latest FU (FU in years)	198 nmol/17 h/mg protein (2)	N.A.	167 nmol/17 h/mg protein (5)	84 nmol/17 h/mg protein (2)
Urinary sulfatide excretion after HCT (FU in years)	N.A.	N.A.	118 nmol/mmol creatinine (5)	235 nmol/mmol creatinine (5)
Brain MRI after HCT at latest FU (FU in years)	Severe global atrophy with stable white matter abnormalities, MLD-LOES score: 8 (9)	Severe global atrophy and diffuse white matter abnormalities with sparing of the U-fibers, MLD-LOES score: 20 (10 months)	Stable extensive white matter abnormalities and mild global atrophy, MLD-LOES score: 16 (5)	Stable extensive white matter abnormalities and global atrophy, MLD-LOES score: 20 (3)
Nerve conduction velocity after HCT (FU in years)				
Ulnar nerve (sensory)	9 m/s (3)	N.A.	No response (1)	25 m/s (2)
Sural nerve (sensory)	N.A.	N.A.	No response (1)	No response (2)
Ulnar nerve (motor)	6 m/s (3)	N.A.	33 m/s (1)	18 m/s (2)
Peroneal nerve (motor)	N.A.	N.A.	28 m/s (1)	15 m/s (2)
Tibial nerve (motor)	5 m/s (3)	N.A.	25 m/s (1)	22 m/s (2)
Distal motor latency after HCT (FU in years)^1^				
Ulnar nerve (motor)	7 ms (3)	N.A.	4 ms (1)	16 ms (2)
Peroneal nerve (motor)	N.A.	N.A.	9 ms (1)	12 ms (2)
Tibial nerve (motor)	21 ms (3)	N.A.	8 ms (1)	5 ms (2)
Survival after HCT (status)	11 years (deceased)	10 months (deceased)	7 years (alive)	3 years (alive)

^1^The sensory and motor median nerve was not assessed in any of the patients

*ARSA* arylsulfatase A gene, *ASA* arylsulfatase A (enzyme), *ATG* anti-thymocyte globulin, *BSID* bayley scales of infant development, *GvHD* graft-versus-host disease, *FU* follow-up after HCT, *HCT* hematopoietic cell transplantation, *HLA* human leukocyte antigen, *IQ* intelligence quotient, *MDI* mental developmental index, *MLD* metachromatic leukodystrophy, *MPNST* malignant peripheral nerve sheath tumor, *MRI* magnetic resonance imaging, *NL* the Netherlands, *WAIS* wechsler adult intelligence scale, *WISC* wechsler intelligence scale for children

In MLD-45, post-HCT complications resulted in delayed tapering of prednisone and replacement of cyclosporine with mycophenolate mofetil (MMF). Her first episode of polyneuropathy progression occurred at 9 months post-HCT when MMF was tapered. A second episode of polyneuropathy progression was observed at 15 months post-HCT after tapering both MMF and prednisone. The differential diagnosis during both episodes included MLD flare-up, neurological GvHD or another (auto)immune-mediated cause, including Guillain–Barre Syndrome (GBS) and chronic inflammatory demyelinating polyneuropathy (CIDP). Brain MRI and cognitive function were stable since the first MRI at 6 months post-HCT showed initial worsening of white matter abnormalities and cerebral atrophy (LOES score increase from 3 to 8). No signs of infection or GvHD of other organs existed. During the first episode, an initial dose of intravenous immunoglobulins (IVIG) did not have clinical effects, but after increasing prednisone dose and restarting MMF a clear clinical improvement was observed. The second episode improved upon a combination treatment of IVIG and MMF. After 13 months, treatment with IVIG was aborted as no further improvement was observed. Treatment with MMF was stopped 2 years later as her polyneuropathy continued to slowly progress. At that time, her brain white matter abnormalities were still stable compared to pre-HCT. Until her passing at 11 years post-HCT, she remained interactive but severely disabled, primarily due to her polyneuropathy. Infrequent spasms suggested the presence of pyramidal symptoms, which were mitigated by her polyneuropathy.

In MLD-50, a slight increase in polyneuropathy was noticed 3 weeks post-HCT, leading to delayed tapering of prednisone with subsequent clinical improvement. A second episode of polyneuropathy progression occurred at 3 months post-HCT after he had a viral infection parallel to decreasing his prednisone dose. Differential diagnoses included MLD flare-up, CMV reactivation, another viral infection, GvHD, or another (auto)immune-mediated cause, including GBS and CIDP. Besides low serum IgG, additional test results were normal. Brain MRI showed white matter abnormalities as expected in the immediate period after treatment (LOES score increase from 2 to 12). Clinical improvement was observed after treatment with one dose of IVIG and increasing prednisone dose. When at 9 months post-HCT prednisone tapering was resumed and another viral infection occurred, he suffered from a third episode of polyneuropathy progression not responding to IVIG, corticosteroids and MMF. Blood examination indicated a mild (viral) infection; lumbar puncture and chest X-ray were negative. Repeated brain MRI showed progression of the white matter abnormalities and severe atrophy (LOES score increase to 20). He died 10 months post-HCT from respiratory failure.

In MLD-62, a severe deterioration of polyneuropathy was observed during the first three post-HCT weeks. The differential diagnosis included GBS, CIDP, neurological GvHD, or MLD flare-up after HCT. Blood examinations were normal, lumbar puncture showed elevated protein and leukocytes. Treatment with methylprednisolone and IVIG resulted in rapid improvement. Two months later, he was hospitalized for multiple respiratory and intestinal viral infections, intestinal GvHD flare-up and pancytopenia. GvHD treatment with MMF was aborted, while cyclosporine tapering was slowed. Another three weeks later, he developed a second episode of rapid polyneuropathy progression and became wheelchair bound within one day. Treatment with IVIG was restarted after three weeks of empiric antibiotic and antiviral therapy and varying dosages of prednisone and hydrocortisone, albeit without immediate clinical effect. Another four weeks later, his motor function showed rapid clinical improvement. Subsequently, his peripheral neuropathy continued to deteriorate gradually, while his brain MRI abnormalities remained stable following an initial increase in the LOES score from 12 to 16 six months post-HCT, with a follow-up of 5.4 years.

**Fig. 1:**
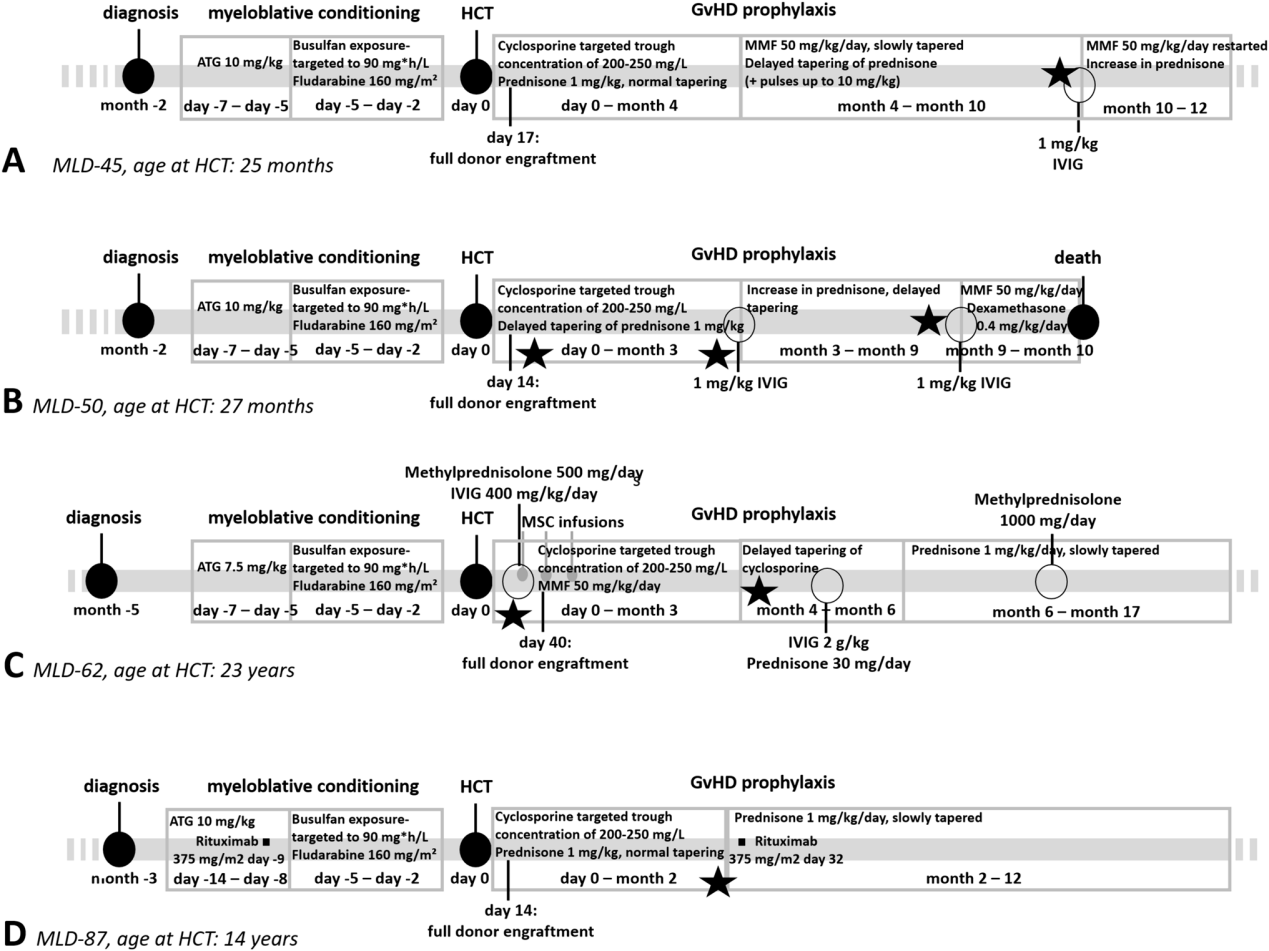
Timeline of immunomodulatory treatments and progression of polyneuropathy. Onset of progression or polyneuropathy is indicated with stars. In MLD-45 **A**, successful engraftment with full-donor chimerism was achieved seventeen days after allogeneic hematopoietic cell transplantation (HCT). Post-HCT complications resulted in delayed tapering of prednisone and replacement of cyclosporine with mycophenolate mofetil (MMF). Polyneuropathy progressed after tapering of immunosuppressive therapy. MLD-50 **B** had successful engraftment with full-donor chimerism fourteen days after HCT. Three weeks after HCT, his polyneuropathy slightly progressed resulting in delayed tapering of prednisone. A second episode of progression of polyneuropathy was treated by one dose of 1 mg/kg intravenous immunoglobulins (IVIG) and an increase in prednisone dose with subsequent clinical improvement. When prednisone tapering was resumed, his polyneuropathy progressed again, eventually leading to death 10 months after HCT. MLD-62 **C** experienced progressive polyneuropathy over three weeks post-HCT that improved rapidly after treatment with methylprednisolone and IVIG. A second episode of rapidly progressive polyneuropathy occurred three weeks after abortion of MMF treatment and slowed tapering of cyclosporine. Treatment with IVIG, in addition to prednisolone treatment of suspected viral retinitis, resulted in no or only limited clinical improvement. Four weeks later, his polyneuropathy rapidly improved after initiating high-dose prednisone treatment of bronchiolitis obliterans syndrome. MLD-87 **D** experienced a severe increase in polyneuropathy 7 weeks post-HCT, after she suffered from a line-associated coagulase negative staphylococcal sepsis. Treatment with prednisolone resulted in full recovery of motor function to pre-HCT level except for an incapacitating tremor of both arms. Her tremor decreased slightly after stopping cyclosporine as GvHD prophylaxis, but almost 3 years after HCT, her tremor is still incapacitating despite treatment with propranolol and levodopa/carbidopa. As HCT conditioning regimen in MLD-87 included rituximab, she required immunoglobulin substitution for 6 months (0.4 g/L IVIG every 3 weeks, followed by immunoglobulins 4 g every week subcutaneously) to maintain serum IgG levels (not shown in figure). *ATG* anti-thymocyte globulin, *GvHD* graft-versus-host disease, *HCT* hematopoietic cell transplantation, *IVIG*: *MMF* mycophenolate mofetil

In MLD-87, a sudden worsening of polyneuropathy was noticed 7 weeks post-HCT, starting with fever from a line-associated coagulase negative staphylococcal bacteremia. Treatment with prednisolone resulted in full recovery of function to pre-HCT level except for an incapacitating tremor of both arms. Although additional test results were suggestive of a Holmes-like tremor, a peripheral origin of the tremor could not be excluded. Her tremor decreased slightly after stopping cyclosporine as GvHD prophylaxis. Treatment effects of propranolol and levodopa/carbidopa were unsatisfying. Nearly three years post-HCT, her tremor remains incapacitating, while her overall motor and cognitive functions, as well as her nerve conduction studies and brain MRI abnormalities—following an initial increase in the LOES score from 11 to 20 within 6 months post-HCT—have remained stable ever since. The timelines of progression of polyneuropathy and immunomodulatory treatments before and in the first year after HCT are shown for all four patients in Fig. 1.

**Fig. 2:**
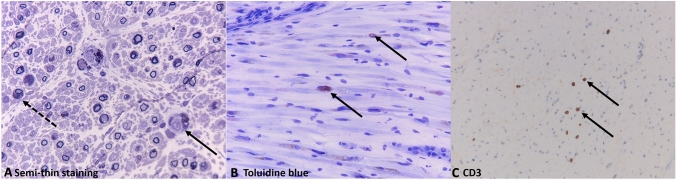
Sural nerve in cross section.** A** Semi-thin staining for electron microscopy shows barely myelinated and unmyelinated axons, myelination of larger axons with thin myelin sheets, signs of secondary axonal degeneration, and macrophages (closed arrow) and Schwann cells (open arrow) loaded with granular material (MLD-50). **B** Toluidine blue staining of the sural nerve reveals only macrophages that contain toluidine blue positive metachromatic material (purple) indicating accumulated sulfatides. No donor macrophages were present (MLD-45).** C** Immunohistochemical staining for CD3 shows the presence of immunopositive T lymphocytes (arrows) in neuronal and surrounding tissue (MLD-45)

In MLD-45 and MLD-50, examination of the sural nerve was performed at the second episode of polyneuropathy progression and postmortem, respectively. Findings are displayed in Fig. 2. Histopathology showed a segmental demyelinating neuropathy with signs of remyelination, secondary axonal degeneration, and sparse perineural and intraneural macrophages and CD3 + T lymphocytes without significant inflammatory infiltration. All macrophages were patient-derived and loaded with sulfatides. No donor macrophages were present in the nerve tissue, in contrast to brain white matter in MLD-50, where presence of donor cells could be confirmed [18]. Patient-derived and donor-derived lymphocytes could not be distinguished, but based on the full-donor chimerism in blood of both patients it was most likely that all tissue lymphocytes were probably donor-derived.

## Discussion

We describe four patients with MLD with significant clinical progression of polyneuropathy after HCT, in parallel with tapering of their immunosuppressive drugs and in three of them with stable brain MRI at the time. Treatment with steroids (prednisone/prednisolone), MMF and IVIG led to partial clinical improvement/stabilization, but not sustained stabilization of polyneuropathy, except for one. The presence of T lymphocytes in the neuronal and surrounding tissue, which is not observed in untreated patients with MLD, in combination with the initial beneficial effects of immunomodulatory drugs suggest a partially (auto)immune-mediated cause of rapid polyneuropathy progression in these transplanted patients with pre-existent MLD induced demyelinating polyneuropathy.

Several MLD cases concurring with or mimicking immune-mediated demyelinating diseases have previously been published, including GBS (*n* = 9) [3, 14, 19–23], CIDP (*n* = 8) [22, 24–28], multiple encephalopathic episodes (*n* = 2) [29, 30] and attacks of acute tumefactive cerebral lesions (*n* = 4) [29, 31–33]. None of these patients had been treated with HCT at the time of these manifestations.

The patients reported here had all been treated with allogenic HCT. Immune-mediated demyelination is a known complication of HCT [15, 16, 34–44]. After HCT, a decrease in immune tolerance while awaiting immune reconstitution may foster the emergence of an immune-mediated demyelinating disease, either induced by auto-reactive patient cells or alloreactive donor-derived cells [15]. In light of the previously published non-transplanted cases [3, 14, 19–33], pre-existing MLD-related damage to the nerves and brain may render individuals with MLD generally more susceptible to (auto)immune-mediated demyelinating diseases. In addition, the higher rate of infections during the immunosuppression period may increase the risk of GBS or CIDP [15].

Immune-mediated polyneuropathies after HCT are typically classified as GBS or CIDP in association with GvHD (GvHD-associated GBS/CIDP) or as GBS or CIDP due to an aberrant immunological response to antecedent infection (classical GBS/CIDP) [16, 35, 37]. Comparable to our patients, symptoms of both GvHD-associated and classical GBS/CIDP often emerge after immunosuppressive drug tapering, and recovery after treatment varies [15, 34–43]. Treatment response is probably dependent on the underlying pathology, with classical GBS/CIDP responding better to IVIG and plasma exchange and GvHD-associated GBS/CIDP to corticosteroids, calcineurin inhibitors, and MMF [37]. Distinction between classical and GvHD-associated GBS/CIDP can, however, be difficult as they share pathological features and GvHD-associated GBS/CIDP may develop when other GvHD manifestations are absent [35]. CSF examination is often normal except for elevated protein level [15, 16, 37, 44], which is common in MLD [8]. Nerve biopsy might be helpful as significant infiltration of donor-derived (alloreactive) CD8 + T lymphocytes would support GvHD-associated GBS/CIDP and its absence classical GBS/CIDP [15]. Nonetheless, classical GBS due to CMV infection after HCT can also be mediated by peripheral expansion of CD8 + T lymphocytes [42]. In addition, an autoimmune mechanism could also be sustained by residual patient’s plasma cells producing autoantibodies and a decrease in immune tolerance [35, 43]. We advise close clinical monitoring of signs of infection and (systemic) GvHD, and careful consideration of repeat electroneurography and possibly peripheral nerve biopsy in these patients. Additionally, the option of repeated skin biopsies for morphological analysis of dermal myelinated nerve fibers could also be considered [45]. Gaining further insight into the progression of peripheral neuropathy may improve tailored therapy in individual cases and enhance our understanding of MLD pathophysiology.

Our patients had a history of (low-grade) acute-GvHD and a temporal relationship between tapering of immunosuppression, worsening of polyneuropathy, and improvement after reinitiating immunosuppression, suggesting GvHD-associated GBS/CIDP. In MLD-45, this was supported by the lack of an infection. Nevertheless, examination of the sural nerve of MLD-45 and MLD-50 did not reveal significant infiltration of probably donor-derived T lymphocytes. Instead, the macrophages present were all patient-derived, suggesting classical GBS/CIDP. Especially classical CIDP may respond to steroids and MMF, and antecedent infections might be absent [16]. Treatment response to IVIG in MLD-45, MLD-62, and MLD-87 although temporarily, additionally supports classical GBS/CIDP as (partial) cause of rapid progression after HCT. In addition, MLD-50 had multiple viral infections and a history of CMV reactivation after HCT, one of the most common viral triggers of GBS. The lack of treatment response to IVIG in this patient might be explained by insufficient dosing based on treating low IgG serum levels and not GBS. In MLD-62, no sural nerve biopsy was performed and the presence of both intestinal GvHD flare-up and several viral infections prior to the progression of polyneuropathy further complicates a retrospective diagnosis. Importantly, his history of type 1 diabetes and blood glucose dysregulation after HCT could have played an additional role for fluctuation of polyneuropathy. Although rapid improvement of peripheral nerve function due to better control of blood glucose levels is unlikely [46], diabetes type 1 is a potential risk factor for CIDP and other autoimmune disorders [16, 47].

Given that sural nerve biopsies revealed no metabolic competent donor macrophages, in contrast to post-HCT brain white matter [18], and only one patient experienced sustained stabilization of polyneuropathy following treatment, it is highly probable that MLD disease progression contributes significantly to the long-term deterioration of polyneuropathy in transplanted patients with MLD, despite stable white matter abnormalities observed on brain MRI. Neither HCT nor immunomodulatory drugs appear to mitigate this progression. Indeed, in late-infantile cases (MLD-45 and MLD-50), rapid polyneuropathy progression aligns with the expected natural course of the disease at their ages [48]. Importantly, such cases would likely not be considered suitable candidates for HCT today due to its inefficacy in late-infantile MLD. Pre-symptomatic late-infantile and pre- and early-symptomatic early-juvenile patients are now eligible for treatment with atidarsagene autotemcel (Libmeldy™, Orchard Therapeutics), an ex vivo autologous hematopoietic stem and progenitor cell-based gene therapy that produces a functional version of the ASA enzyme at supra-physiological concentrations. Fumagalli et al*.* demonstrated that late-infantile patients treated with atidarsagene autotemcel exhibited significantly improved nerve conduction velocities compared to age-matched natural history control patients at 2 and 3 years post-treatment, although results in early-juvenile patients were variable [49]. Notably, no cases of rapid polyneuropathy progression shortly after treatment with atidarsagene autotemcel have been documented thus far, indicating the potential for genetically modified blood cells to migrate into the peripheral nervous system and differentiate into ASA-producing endoneural macrophages. Alternatively, the elevated levels of ASA achieved by the therapy may facilitate enzyme penetration of the peripheral nerves [49].

The typical course of polyneuropathy progression in late-juvenile and adult MLD generally remains stable [48]. However, it is plausible that polyneuropathy progression was triggered post-HCT, akin to the previously documented progression of white matter abnormalities and atrophy on brain MRI within the first year post-treatment [10]. HCT-related drug toxicity, particularly cyclosporine, may have additionally contributed to the rapid progression of polyneuropathy in all patients. Nonetheless, it’s noteworthy that most drug-induced toxic neuropathies are axonal in origin and tend to improve rather than worsen following cessation of the offending drug [16].

An important limitation of our study is the lack of a definitive cause for peripheral neuropathy. This emphasizes the challenge of characterizing progressive polyneuropathy in HCT-treated patients with MLD and the need for additional research. Another limitation is that, due to the retrospective study design, our findings rely on the accuracy of the patient records and on the performed tests and treatments in a clinical care setting, e.g., no nerve conduction study at time of clinical polyneuropathy progression was performed in MLD-50 and MLD-62. In addition, no nerve biopsy was performed in MLD-62 and MLD-87, and it is uncertain if the observed T lymphocytes in the biopsies of MLD-45 and MLD-50 are donor- or patient-derived, although the latter is less likely considering the short lifespan of T lymphocytes and full-donor chimerism in blood of all patients achieved months before examination. Besides this, it is impossible to establish cause and effect. The goal of this paper was to gain more knowledge on progressive polyneuropathy shortly after HCT. Therefore, we did not compare patients with and without progression of polyneuropathy following HCT.

Lastly, it is important to highlight that the long-term disability observed in our patients was influenced by the involvement of both the central and peripheral nervous systems. Similar to the cases of patients MLD-4 and MLD-37 described by Al-Saady et al. [50], we observed signs of slowly progressive pyramidal symptoms in MLD-45 and MLD-62, as well as gradual cognitive decline in MLD-62 during long-term follow-up.

In conclusion, characterizing progressive demyelinating neuropathy, as occurring in some MLD patients treated with HCT, is challenging. Why central and peripheral myelin behave differently in response to HCT, is not understood. This knowledge gap needs to be filled to help physicians making the correct diagnosis and selecting the most effective treatment. Severe peripheral neuropathy is immensely debilitating. The findings in our transplanted patients with rapid polyneuropathy progression parallel with tapering of their immunosuppressive drugs suggest a partially (auto)immune-mediated pathology. Therefore, it might be considered to add or increase immunomodulatory drugs in patients with MLD and progressive polyneuropathy following HCT, especially during periods of suspected immune activation such as infection, and GvHD.

### Supplementary Information

Below is the link to the electronic supplementary material.

Supplementary file1 (DOCX 43 KB)

## Data Availability

Not applicable.
