# Supplementary material for: Progressive demyelinating polyneuropathy after hematopoietic cell transplantation in metachromatic leukodystrophy: a case series
Source: J Neurol. 2024 Apr 2;271(7):4028–38. doi: 10.1007/s00415-024-12322-3 (PMC11233286; doi:10.1007/s00415-024-12322-3)
Supplement: Supplementary file 1 — Supplementary file1 (DOCX 43 KB) [file 415_2024_12322_MOESM1_ESM.docx]

# Supplementary data

## **MLD-45**

MLD-45 has been described previously (Squint-4) [1]. In short, this girl with late-infantile MLD was referred for treatment evaluation at the age of 24 months. Neurological examination revealed slight tremor, mild dysarthria, wide-based gait, and absent deep tendon reflexes. Nerve conduction studies demonstrated a severe demyelinating sensorimotor polyneuropathy. Cognitive tests were normal and brain MRI showed subtle myelination delay and mild nonspecific white matter abnormalities. HCT was performed at the age of 25 months. Patient characteristics, test results and treatment details before and after HCT are presented in **Table 1**.

Post-HCT course was complicated by slow restitution of T lymphocytes (<50 CD4+ T cells/μL six months post-HCT [2]), mild acute-GvHD grade one with skin involvement, multiple viral upper respiratory tract infections, and cyclosporine-induced pericarditis. The latter two complications resulted in delayed tapering of prednisone and replacement of cyclosporine with MMF, respectively. Six months after HCT, neurological examination and brain MRI —following an initial increase in the LOES score from 3 to 8 — had stabilized. However, when MMF was tapered in the following months, polyneuropathy aggravated, resulting in progressive generalized limb weakness, axial hypotonia and severe ptosis. In addition, increased tremor and ataxia were noticed. Cognitive development and brain white matter abnormalities were stable. There was no evidence of infection or GvHD. Immune serology showed low IgG which was treated with one dose of 1 mg/kg intravenous immunoglobulins (IVIG) without clinical improvement. Prednisone dose was increased (daily 1 mg/kg) and MMF was restarted (daily 50 mg/kg). Subsequently, her symptoms improved. The timeline of immunomodulatory treatments before and after HCT and progression of polyneuropathy is shown in **Figure 1A**.

Six months later, her paresis and ptosis increased again after tapering MMF and prednisone. Nerve conduction studies confirmed progression of demyelinating polyneuropathy, supported by a decrease in tibial nerve NCV from 16 m/s to 4.6 m/s and an increase in distal motor latency from 9 m/s to 20.6 m/s. No response was elicited upon measuring the facial nerve. The differential diagnosis included MLD flare-up, neurological GvHD or another (auto)immune-mediated cause. Brain MRI was stable. Neither clinical nor laboratory-confirmed signs of infection or GvHD of other organs existed. Serological antibody tests for myasthenia gravis were negative. Biopsy of the sural nerve **(Figure 2)** showed a segmental demyelinating neuropathy with signs of remyelination, secondary axonal degeneration, and sparse perineural and intraneural macrophage and CD3+ T lymphocyte presence without significant inflammatory infiltration. All macrophages were patient-derived and loaded with sulfatides. Treatment with IVIG (daily 400 mg/kg Nanogam for five days, then every five weeks) and MMF (daily 25 mg/kg) for presumptive chronic inflammatory demyelinating polyneuropathy (CIDP) was initiated with subsequent clinical improvement. After 13 months, IVIG treatment was aborted as no further improvement was observed. Two years later, MMF was stopped because of recurrent agranulocytosis and mild progression of her polyneuropathy. At that time, her brain white matter abnormalities were still stable compared to pre-HCT. Ten years after HCT, she was alert and interactive but unable perform any voluntary movements and with a severe ptosis. Infrequent spasms suggested the presence of pyramidal symptoms, which were mitigated by her polyneuropathy. Infrequent spasms suggested the presence of pyramidal symptoms, which were mitigated by her polyneuropathy. At the age of thirteen years, she died from a respiratory infection.

## MLD-50

Presentation and treatment of MLD-50 with late-infantile MLD was previously described (patient 1) [3]. Neurological examination at the age of 25 months showed a slight tremor, reduced tone and strength of proximal and distal muscles, mild atrophy of the hypothenar and interossei muscles, and diminished deep tendon reflexes. Nerve conduction studies confirmed a severe demyelinating sensorimotor polyneuropathy. Cognitive function was age adequate, and brain MRI showed a subtle myelination delay. HCT was performed at the age of 27 months (**Table 1**).

Post-HCT course was complicated by acute-GvHD grade one with skin involvement and mucositis, and cytomegalovirus (CMV) reactivation. Three weeks after HCT, a bilateral nystagmus and slight reduction in muscle strength of the limbs, distally more than proximally, was noticed, leading to delayed tapering of prednisone. His muscle strength increased the following two months, but after a viral infection at the same time as prednisone was tapered to 0.1 mg/kg twice daily, he started to have episodes of fever, vomiting, fluctuating muscle weakness, and reduced exercise tolerance. Differential diagnoses included MLD flare-up, CMV reactivation, another viral infection, GvHD, or another (auto)immune-mediated cause. Immune serology showed low IgG which was treated with one dose of 1 mg/kg IVIG. Other laboratory examinations, including viral titers, chest X-ray and cardiac echocardiography were normal. Prednisone dose was increased (0.6 mg/kg daily) with subsequent clinical improvement. The timeline of immunomodulatory treatments before and after HCT and progression of polyneuropathy is presented in **Figure 1B**. MRI at six months post-HCT revealed white matter abnormalities with sparing of the U-fibers as expected in the immediate period after treatment (LOES score increase from 2 to 12). Nine months after HCT, when prednisone tapering was resumed and another viral infection occurred, his muscle strength diminished and he could no longer sit without support. Examination showed an increase in cerebellar symptoms and polyneuropathy. A second dose of 1 mg/kg IVIG was given. Prednisone was replaced by dexamethasone (0.4 mg/kg daily) and MMF (50 mg/kg daily), but his muscle strength further decreased. Lumbar puncture and chest X-ray showed no signs of infection, but his blood showed increased level of C-reactive protein (30 mg/L) and leukocytosis (20x10^9/L). Repeated brain MRI showed progression of the white matter abnormalities and severe atrophy (LOES score increase to 20). He died two weeks later from respiratory failure. and he died two weeks later from respiratory failure. Postmortem analysis of the sural nerve (**Figure 2**) showed barely myelinated and unmyelinated axons, signs of secondary axonal degeneration, few perineural and intraneural CD3+ T lymphocytes, and patient-derived macrophages loaded with sulfatides. No donor macrophages were present in the tissue while those were observed in the brain white matter [3].

## MLD-62

MLD-62, a male with a history of poorly controlled type I diabetes since the age of 4 years, was referred at the age of 23 years after family testing revealed adult MLD. From one year before diagnosis, he experienced gradually progressive numbness and pain in his feet. At pre-HCT assessment, he had a mild foot drop and diminished deep tendon reflexes. Cognitive function was normal. Nerve conduction studies confirmed a severe demyelinating sensorimotor polyneuropathy. Brain MRI showed bilateral white matter abnormalities with typical sparing of the U-fibers and mild cerebral atrophy. HCT was performed four months after diagnosis (**Table 1**).

Post-HCT course was complicated by 1) diabetes dysregulation; 2) syndrome of inappropriate antidiuretic hormone secretion; 3) staphylococcus epidermidis sepsis; 4) line-associated thrombosis; 5) clostridium intestinal infection; and 6) acute-GvHD grade 3 with intestinal involvement that needed treatment with prednisolone and three infusions of mesenchymal stromal cells. In addition, he experienced severely progressive sensory impairment and muscle weakness, distal more than proximal, over the first three post-HCT weeks. The differential diagnosis included Guillain-Barre Syndrome (GBS), CIDP, neurological GvHD, or MLD flare-up after HCT. Routine laboratory investigations, serum cultures, and viral antibody titers were negative. Lumbar puncture showed elevated protein (1.02 g/L) and leukocytes (25x10^6/L). Pragmatic treatment with five days methylprednisolone (8 mg/kg daily) and IVIG (400 mg/kg daily) in addition to treatment of his other complications resulted in rapid improvement of his sensory and motor function. The timeline of immunomodulatory treatments before and after HCT and progression of polyneuropathy is presented in **Figure 1C**. Two months later, he was hospitalized for multiple respiratory and intestinal viral infections (respiratory syncytial virus, adenovirus, and norovirus) in combination with intestinal GvHD flare-up and pancytopenia. GvHD treatment with MMF was aborted, while cyclosporine tapering was slowed. Another three weeks later, he developed rapidly progressive sensory and motor loss and became wheelchair bound within one day. Examination showed global muscle atrophy, reduced muscle strength and loss of sensory function, most prominent in the legs, with absent deep tendon reflexes. Serological antibody tests for Lambert-Eaton myasthenic syndrome were negative. Treatment with IVIG (2 g/kg over three days) was restarted for presumptive CIDP after three weeks of empiric antibiotic and antiviral therapy and varying dosages of prednisone and hydrocortisone, albeit without immediate clinical effect. In addition, prednisolone (0.5 mg/kg/day for 7 days) was started as treatment of suspected viral retinitis, followed by a slowed tapering regimen. Four weeks later, prednisone treatment (1 mg/kg/day) of bronchiolitis obliterans syndrome was initiated. From then, his sensorimotor functions rapidly improved, enabling him to walk without support. Nerve conduction study results were also improved at later examination. However, his sensorimotor functions continued to deteriorate gradually, accompanied by slowly progressive pyramidal signs and cognitive decline in addition to peripheral neuropathy at neurological examination. Despite this decline, he remains capable of walking indoors without assistance. . Brain MRI showed initial progression of white matter abnormalities and mild atrophy at six months after HCT (LOES score increase from 12 to 16) but has been stable ever since with a follow-up of 5.4 years.

## MLD-87

MLD-87 is a female with late-juvenile MLD and a history of general clumsiness and slightly reduced fine motor skills. In addition, she was diagnosed with a malignant peripheral nerve sheath tumor (MPNST) at the age of 12 years, that was successfully treated with high-dose doxorubicin and ifosfamide (cumulative doses doxorubicin: 225 mg/m2, ifosfamide: 36 g/m2), surgical resection and brachytherapy. She was referred at the age of 13 years with coordination and balance difficulties, stress urinary incontinence with sometimes involuntary soiling, and a tremor of both arms since her tumor treatment. Neurological exam revealed a mild foot drop, tandem gait difficulty (missteps), a mild intention tremor, mild hypertonia of the lower limbs, slightly reduced muscle strength of upper and lower limbs, decreased deep tendon reflexes, and bilateral Babinski signs. Cognitive function was below average without evidence of decline. Nerve conduction studies indicated a demyelinating sensorimotor polyneuropathy with strongly reduced conduction velocities and additionally conduction blocks in the median and peroneal nerves. Nerve ultrasound revealed diffuse and focal nerve enlargement. Brain MRI showed bilateral white matter abnormalities with typical sparing of the U-fibers and mild cerebral and cerebellar atrophy. HCT was performed three months after diagnosis (**Table 1**). As her HCT conditioning regimen included rituximab, she required immunoglobulin substitution for 6 months (0.4 g/L IVIG every 3 weeks, followed by 4 g immunoglobulins every week subcutaneously) to maintain serum IgG levels.

Post-HCT course was complicated by line-associated coagulase negative staphylococcal sepsis, after which she experienced an incapacitating tremor of both arms, articulation disorder, increased paresis of all limbs leading to the inability to walk, and areflexia at 7 weeks after HCT (**Figure 1D**). Except for her tremor, her function fully recovered to pre-HCT level after treatment with prednisone (1 mg/kg). The tremor was present in the proximal muscles of both arms with a frequency at 4 Hz at rest, exacerbated with posture and additionally intensified with action. It showed an intermittent left and right predominance and there was no coherence between left and right. Making a ballistic movement resulted in some tremor inhibition with simultaneous movement of the outstretched arm, suggestive of a Holmes-like tremor, although a peripheral origin of the tremor could not be excluded. Other neurological examination was comparable to pre-HCT. Brain MRI revealed initial progression of white matter abnormalities, and cerebral and cerebellar atrophy at six months after HCT (LOES score increase from 11 to 20). Nerve conduction studies showed a stable demyelinating polyneuropathy with persisting conduction blocks in the left peroneal nerve (comparable to pre-HCT), and a few compound muscle action potential (CMAP) amplitudes that were higher than pre-HCT, possibly influenced by variances in age and physical resilience. Her tremor decreased slightly after stopping cyclosporine as GvHD prophylaxis. Treatment with propranolol (20 mg three times daily) had no effect on the tremor. Treatment with levodopa/carbidopa (50/12.5 mg three times daily) resulted in minimal improvement. Almost 3 years after HCT, her tremor is still incapacitating while her overall motor and cognitive function as well as her white matter abnormalities on brain MRI remained stable ever since.

1. Beerepoot, S., et al., *Acute-onset paralytic strabismus in toddlers is important to consider as a potential early sign of late-infantile Metachromatic Leukodystrophy.* Eur J Paediatr Neurol, 2022. **37**: p. 87-93.

2. Boelens, J.J., K.K. Hosszu, and S. Nierkens, *Immune Monitoring After Allogeneic Hematopoietic Cell Transplantation: Toward Practical Guidelines and Standardization.* Front Pediatr, 2020. **8**: p. 454.

3. Wolf, N.I., et al., *Metachromatic leukodystrophy and transplantation: remyelination, no cross-correction.* Ann Clin Transl Neurol, 2020. **7**(2): p. 169-180.
